# Salinity impairs photosynthetic capacity and enhances carotenoid-related gene expression and biosynthesis in tomato (*Solanum lycopersicum* L. cv. Micro-Tom)

**DOI:** 10.7717/peerj.9742

**Published:** 2020-09-17

**Authors:** Andrés Leiva-Ampuero, Mario Agurto, José Tomás Matus, Gustavo Hoppe, Camila Huidobro, Claudio Inostroza-Blancheteau, Marjorie Reyes-Díaz, Claudia Stange, Paulo Canessa, Andrea Vega

**Affiliations:** 1 Millennium Institute for Integrative Biology (iBio), Santiago, Chile; 2 Departamento de Genética Molecular y Microbiología, Facultad de Ciencias Biológicas, Pontificia Universidad Católica de Chile, Santiago, Chile; 3 Facultad de Agronomía e Ingeniería Forestal, Pontificia Universidad Católica de Chile, Santiago, Chile; 4 Centro de Biotecnología Vegetal, Facultad de Ciencias de la Vida, Universidad Andres Bello, Santiago, Chile; 5 Institute for Integrative Systems Biology, I^2^SysBio, Universitat de València - CSIC, Valencia, Spain; 6 Núcleo de Investigación en Producción Alimentaria (NIPA), Departamento de Ciencias Agropecuarias y Acuícolas, Facultad de Recursos Naturales, Universidad Católica de Temuco, Temuco, Chile; 7 Departamento de Ciencias Químicas y Recursos Naturales, Facultad de Ingeniería y Ciencias, Universidad de La Frontera, Temuco, Chile; 8 Center of Plant, Soil Interaction, and Natural Resources Biotechnology, Scientific and Technological Bioresource Nucleus (BIOREN), Universidad de La Frontera, Temuco, Chile; 9 Centro de Biología Molecular Vegetal (CBMV), Departamento de Biología, Facultad de Ciencias, Universidad de Chile, Santiago, Chile

**Keywords:** Abiotic stress, Photosynthesis, Salt stress, Tomato fruits, Lycopene

## Abstract

Carotenoids are essential components of the photosynthetic antenna and reaction center complexes, being also responsible for antioxidant defense, coloration, and many other functions in multiple plant tissues. In tomato, salinity negatively affects the development of vegetative organs and productivity, but according to previous studies it might also increase fruit color and taste, improving its quality, which is a current agricultural challenge. The fruit quality parameters that are increased by salinity are cultivar-specific and include carotenoid, sugar, and organic acid contents. However, the relationship between vegetative and reproductive organs and response to salinity is still poorly understood. Considering this, *Solanum lycopersicum* cv. Micro-Tom plants were grown in the absence of salt supplementation as well as with increasing concentrations of NaCl for 14 weeks, evaluating plant performance from vegetative to reproductive stages. In response to salinity, plants showed a significant reduction in net photosynthesis, stomatal conductance, PSII quantum yield, and electron transport rate, in addition to an increase in non-photochemical quenching. In line with these responses the number of tomato clusters decreased, and smaller fruits with higher soluble solids content were obtained. Mature-green fruits also displayed a salt-dependent higher induction in the expression of *PSY1*, *PDS*, *ZDS*, and *LYCB*, key genes of the carotenoid biosynthesis pathway, in correlation with increased lycopene, lutein, β-carotene, and violaxanthin levels. These results suggest a key relationship between photosynthetic plant response and yield, involving impaired photosynthetic capacity, increased carotenoid-related gene expression, and carotenoid biosynthesis.

## Introduction

In plants, salt stress can be considered as an environmental restriction consisting of an osmotic or water-deficit and a salt-specific or ion-excess effect. In both cases, salt stress interferes with the plant’s nutrient balance, negatively affecting growth and development. Salt stress also hampers photosynthetic capacity, impairing CO_2_ supply, stomatal conductance, transpiration, and thus alters photosynthetic carbon assimilation and associated metabolism, among other physiological effects (Flexas et al., 2004; Munns, 2005; Munns, James & Läuchli, 2006; Chaves, Flexas & Pinheiro, 2009; Yang & Guo, 2018a; Yang & Guo, 2018b). As a consequence, the generation of reactive oxygen species (ROS) and the accumulation of toxic compounds cause secondary oxidative stress (Hernández et al., 2012; Yang & Guo, 2018b). In response to salinity, plants utilize an array of biochemical and physiological mechanisms to counteract the detrimental effects, involving changes in the expression of genes associated with the biosynthesis of osmoprotectant molecules, detoxifying enzymes, late-embryogenesis-abundant proteins, and phytohormone biosynthesis (Munns, 2005; Kumari et al., 2009; Rajendran, Tester & Roy, 2009; Yao et al., 2011), among others.

Tomato (*Solanum lycopersicum* L.) is a plant moderately tolerant to salinity at all developmental stages (Maas, 1986; Jones, Hashim & El-Beltagy, 1988). Tomato plants exposed to high levels of salt (i.e., 70 mM NaCl) display a decrease in seed germination and an alteration in the absorption of water by roots (Cuartero & Fernández-Muñoz, 1999), as well as a reduction in shoot growth, stomatal density, leaf number and area (Li et al., 1999; Romero-Aranda, Soria & Cuartero, 2001). Moreover, salinity induces leaf senescence and increases the root/shoot ratio (Ghanem et al., 2008; Albacete et al., 2008). Photosynthesis is also reduced under salt stress in this species, displaying, for example, a reduction of maximum efficiency of PSII photochemistry and alterations in the size and number of active reaction centers of the photosynthetic apparatus, as well as the quantum yield of primary photochemistry and electron transport (Moles et al., 2016; Singh, Singh & Prasad, 2016). Although salinity negatively affects the normal development of vegetative tissue in tomato plants, it has been reported that salt stress improves fruit quality by increasing sugar and organic acids contents, while also increasing the carotenoid levels, thereby improving the nutritional value (Adams & Ho, 1989; Adams, 1991; De Pascale et al., 2001; Wu, Buck & Kubota, 2004; Wu & Kubota, 2008; Borghesi et al., 2011; Massaretto et al., 2018). Studies in tomato landraces indicated that salinity improve fruit quality attributes, including flavonoids and sugar (Moles et al., 2019).

Nevertheless, some studies indicate that salinity had only a marginal effect on carotenoid concentration in tomato fruit (Petersen, Willumsen & Kaack, 1998; Krauss et al., 2006; Van Meulebroek et al., 2012; Van Meulebroek et al., 2016). These conflicting findings can be explained, at least in part, by differences in the genetic background of the tomato cultivars and their associated salt tolerances (Sun et al., 2010; Martínez et al., 2014), the timing and the degree of salt treatment (Mizrahi et al., 1988; Dorais, Ehret & Papadopoulos, 2008), and other environmental factors observed in greenhouses and productive environments (Cuartero & Fernández-Muñoz, 1999; Dumas et al., 2003; Ehret et al., 2013). This evidence indicates that the relationship between salinity and tomato fruit carotenoid content is complex, requiring in-deep investigation. Moreover, the molecular mechanisms behind the enhanced carotenoid concentration under high salinity conditions remain unknown.

In plants, carotenoids are lipid-soluble pigments found in cell membranes of chloroplasts and chromoplasts. These pigments are essential structural components of the antenna complex, acting as auxiliary light-collecting pigments and photoprotectors of photosynthetic apparatus against excess light (Frank & Cogdell, 1996; Havaux, 1998), precursors of abscisic acid (Schwartz, Qin & Zeevaart, 2003), and also contributing to coloration in fruits and flowers (Bartley & Scolnik, 1995). In tomato, lutein is the main carotenoid present in leaves and acts as a photoprotector (Standfuss et al., 2005). Meanwhile, xanthophylls, violaxanthin, and neoxanthin are mostly present in flowers, conferring their yellow coloration (Galpaz et al., 2006). Lycopene is the main carotenoid found in fruits and is responsible for the characteristic red color observed at the ripening stage (Fraser et al., 1999). Also, different isoprenoid intermediates can be found in tomato fruits at lower concentrations, such as the colorless phytoene and phytofluene, the orange-color β-carotene, and also γ-carotene, δ-carotene, lutein, neurosporene, and α-carotene (Fray & Grierson, 1993; Galpaz et al., 2006; Slimestad, Fossen & Verheul, 2008). Carotenoids play a role in increasing the nutritional quality of tomato fruit, providing health-related benefits (Fraser & Bramley, 2004; Ruiz-Sola & Rodríguez-Concepción, 2012).

The type of carotenoid present in different plant tissues, as well as its levels, are highly conditioned by the genetic background, agronomic setting, and environmental conditions during plant growth (Tiwari & Cummins, 2013). Notwithstanding that enhanced carotenoid concentration in response to salinity is a cultivar-specific trait, most salt-resistant cultivars display a greater synthesis of carotenoids (Juan et al., 2005; Coesel et al., 2008), which suggests a potential protective role of these compounds against salinity-related impairment of plant fitness. Little is known about the trade-off between salinity-triggered reductions in photosynthesis capacity, which is concomitant with a decrease in yield, and the salinity-dependent increase of carotenoids, improving, at least in part, fruit quality. In this work, we characterized the effect of a gradient of salinity conditions in *S. lycopersicum* cv. Micro-Tom, regarding photosynthesis capacity and parameters associated with yield and fruit quality, with emphasis on carotenoid biosynthesis. To determine the relationship between plant photosynthetic capacity and carotenoid biosynthesis in response to saline stress in this model plant species, plants were long-term exposed to different NaCl levels, evaluating photosynthetic parameters, carotenoid accumulation, as well as the expression of key genes of their biosynthesis pathway during two developmental stages.

## Materials & Methods

### Plant material, growth conditions and salt treatments

*Solanum lycopersicum* cv. Micro-Tom seeds were imbibed at 4 °C in darkness for 48 h and then sown in plates with Murashige and Skoog culture medium (0.5×). Plates were placed in a growth chamber under controlled conditions for 1 week at 60–80% relative humidity, 24 ± 1 °C, 16/8 h light/dark photoperiod, and 120 mol m^−2^ s^−1^ of light intensity. Thereafter, 7-day-old seedlings were transferred into pots with a peat and vermiculite mixture (2:1), irrigated twice per week with deionized water, and fertilized with 0.5× Hoagland solution (Sigma-Aldrich) for 120 days under the same environmental conditions.

For salt treatments, plants with five to six leaves were irrigated twice per week with 0, 40, 80, 120, and 160 mM NaCl solutions (100 ml of each salt solution per plant individually cultivated in 300 cm^3^ pot) in addition to Hoagland fertilization, for 14 weeks (until harvest). The experimental design was randomized into a complete block with at least three biological replicates for each treatment.

### Plant sampling and phenotypic analysis

Vegetative and reproductive organs of tomato plants exposed to continuous NaCl irrigation were evaluated for 14 weeks. Leaves samples were taken at 2 and 8 weeks of salinity treatment (wst) for analyzing photosynthetic parameters. Fruit analyses were carried out at 11 and 14 wst, which represent the mature-green (at 40 days post-anthesis (dpa)) and red-ripe (at 60 dpa) developmental stages, respectively. For phenotypic and physiological analysis of fruits, the number of clusters, fresh weight, caliber, and total soluble solids were measured. Fruits were also evaluated using the CIEL*a*b* system with a colorimeter that measured L*, a*, and b* values. Briefly, colors are defined by an achromatic component L*, representing relative darkness or lightness, and the chromatic descriptors a* and b*, indicating the red and yellow color intensity, respectively (CIE, 2004).

### Photosynthetic parameters

Net photosynthesis (Pn) and stomatal conductance (gs) were measured in the first and second leaves of three randomly selected plants exposed to 2 and 8 wst, using a portable CO_2_ infrared gas analyzer (Licor LR6400, Lincoln, NE, USA), equipped with a cuvette that controlled irradiance (600 µmol photons m^−2^s^−1^), temperature (20 °C), humidity (80%), CO_2_ concentration (400 µmol mol^−1^), and flow rate (300 cm^3^ min^−1^).

Leaf chlorophyll fluorescence was measured with a portable pulse amplitude modulated fluorimeter (FMS 2; Hansatech Instruments, Norfolk, UK), in the same leaves used for measuring photosynthetic parameters and previously exposed to darkness for 20 min. Maximum quantum yield of PSII (Fv/Fm), effective quantum yield of PSII (ΦPSII), and electron transport rate (ETR) were estimated according to Genty, Briantais & Baker (1989), whereas non-photochemical quenching (NPQ) was estimated as described by Maxwell & Johnson (2000).

### Carotenoid levels analysis

Carotenoids were extracted from 5 g of tomato fruits, considering a mixture of exocarp, mesocarp and endocarp (pericarp of the fruit). Samples were homogenized in a mortar with 1 g of quartz sand and 0.5 g of ascorbic acid. The initial extraction procedure was carried out according to Biacs & Daood (1994), followed by the extraction of carotenoids with 1,2-dichloroethane. Phase and carotenoid separations, as well as carotenoid quantification by high-performance liquid chromatography with diode array detector (HPLC-DAD), were performed as described by Daood et al. (2014). Total carotenoids were measured by spectrophotometry at 474 nm. Specific carotenoids were analyzed using HPLC-DAD equipped with a Hiperst C18 column (Merck). This system was operated with a mobile phase (1 ml min^−1^), acetonitrile: methanol: methylene chloride (85:10:5 v/v) at room temperature with isocratic conditions. The carotenoids were identified according to their absorption spectra, retention time and comparison with specific pigment standards. Carotenoid levels were expressed in lg/g of dry weight (lg/g DW). Extractions were performed and analyzed in triplicate, on ice and dark conditions. The conversion factor for fresh to dry weight in each experimental condition was determined by desiccation of 1g of fresh weight in an oven at 65 °C for 72 h.

### Gene expression analysis

Fruits belonging to the first and second clusters were sampled from each plant, and total RNA was extracted using a CTAB-Spermidine method as described in Reid et al. (2006). RNA was treated with RQ1 DNAse (Promega) and quantified by spectrophotometry using a NanoDrop® ND-1000 spectrophotometer (Thermo Scientific™). For cDNA synthesis, RNA was reversely transcribed using oligo(dT)25 and RevertAid Reverse Transcriptase (Thermo Fisher™), following the manufacturer’s instructions.

The reverse-transcription quantitative real-time PCR (RT-qPCR) analysis was performed using the SensiMix™ Plus SYBR commercial kit (Quantace) and the StepOne™ Real-Time PCR System (Applied Biosystems™, Foster City, CA, USA). Primers for the analyzed carotenoid-pathway genes (Table S1) were designed with the Primer-BLAST Tool (https://www.ncbi.nlm.nih.gov/tools/primer-blast/). Relative gene expression levels were calculated using the ΔΔC_T_ method (Livak & Schmittgen, 2001), employing three biological replicates. For mRNA levels normalization, the *actin gene* (Solyc04g011500.3) was used. The expression of this reference gene was stable in our experimental conditions.

### Statistical analysis

The data presented in this study are shown as the mean ± SE (*n* = 3 − 16) for each treatment, depending on the evaluated variable. Differences between treatments were assessed using one-way analysis of variance (ANOVA) followed by Tukey’s multiple comparison test, considering *P* ≤ 0.05.

## Results

### Photosynthetic capacity is hampered by salt stress in tomato plants

**Figure 1:**
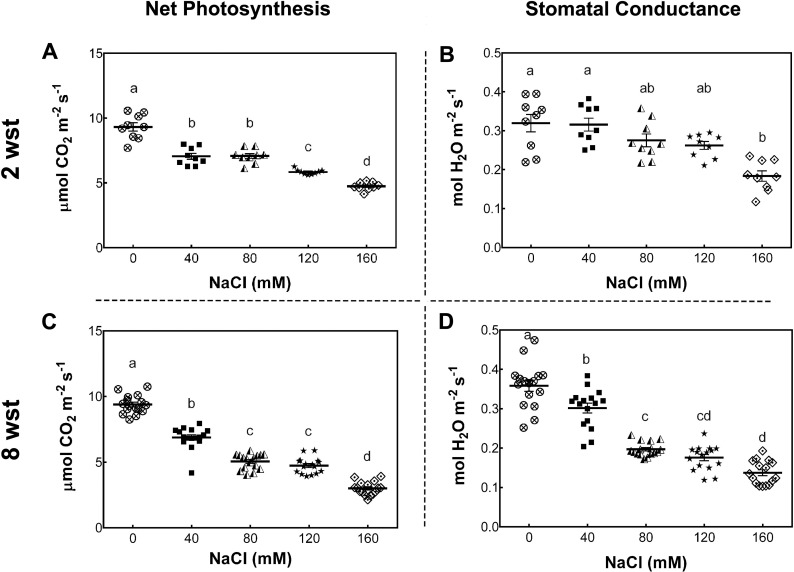
Net photosynthesis and stomatal conductance in salt-stressed plants. Samples were collected at the same time of the day at 2 and 8 weeks after salinity treatment (wst) with 0, 40, 80, 120, or 160 mM NaCl irrigation. Data points represent mean ± SEM considering nine and sixteen biological replicates for 2 (A, B) and 8 wst (C, D). Different letters indicate statistically significant differences among treatments as determined by Tukey’s multiple comparison test (*P* ≤ 0.05).

To determine the impact of long-term salt-stress on plant growth and fruit development, 3-week-old *S. lycopersicum* cv. Micro-Tom plants were exposed to saline irrigation twice per week with 0, 40, 80, 120, and 160 mM NaCl solutions (see Methods). Foliar photosynthetic parameters were evaluated after 2 and 8 weeks of salinity treatment (wst). The net photosynthesis (Pn) rate (as indicated by CO_2_ assimilation) decreased with the increase in salt concentration at both sampling dates (Figs. 1A, 1C). Since Pn reduction is known to be partly due to a reduced stomatal conductance (gs), as described in several plant species (Munns, James & Läuchli, 2006; Chaves, Flexas & Pinheiro, 2009), we also determined gs under our conditions. At 2 wst, plants showed a 43% reduction in gs only at 160 mM NaCl, while at 8 wst a decreased gs was observed from 40 mM salt treatment onwards, reaching a 63% reduction at the highest salt-treatment concentration (Figs. 1B, 1D).

**Figure 2:**
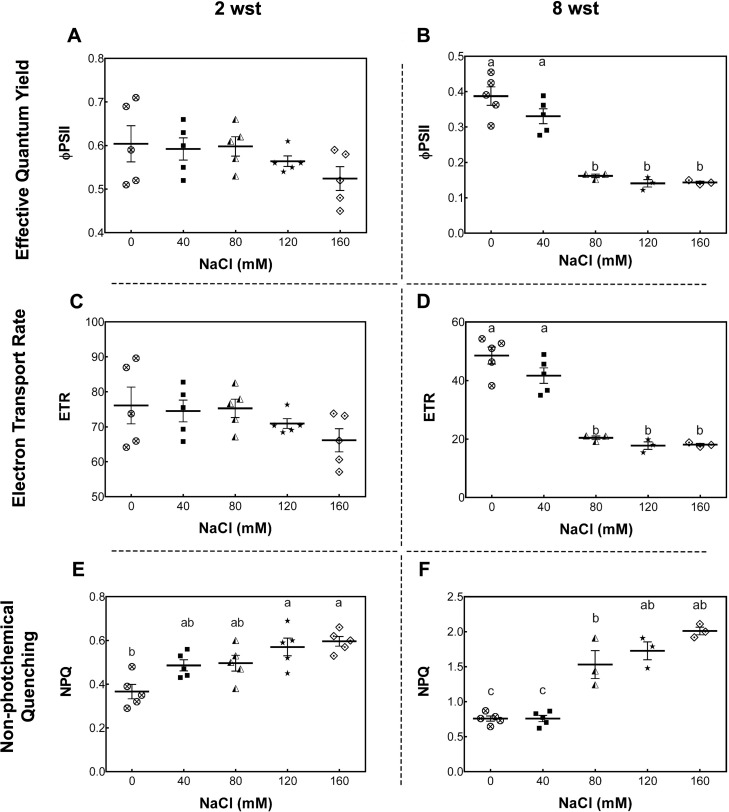
Leaf chlorophyll fluorescence parameters in salt-stressed plants. Samples were collected at 2 (A, C, E) and 8 weeks (B, D, F) of 0, 40, 80, 120, or 160 mM NaCl irrigation. Data points represent mean ± SEM of five and between three and five biological replicates for 2 and 8 weeks of salinity treatment (wst), respectively. Different letters indicate statistically significant differences among treatments, as determined by Tukey’s multiple comparison test (*P* ≤ 0.05).

To further evaluate the damage of salt stress in the photosynthetic apparatus, maximum quantum yield of PSII (Fv/Fm), effective quantum yield of PSII (ΦPSII), electron transport rate (ETR), and non-photochemical quenching (NPQ) were measured. For Fv/Fm no significant differences between treatment and time were observed (Fig. S1). At 2 wst, ΦPSII and ETR in all salt-treated plants were similar to those in the non-saline control treatment (Figs. 2A and 2C). However, our NPQ analysis shows that 120 and 160 mM NaCl lead to a significant increase of NPQ compared to control plants (Fig. 2E). On the other hand, significant differences were observed at 8 wst in all three measured parameters from 80 mM NaCl upwards (Figs. 2A, 2C and 2E), indicating that the exposure time is an important factor modulating the response to salinity.

Because salt-stress treatments were imposed before the flowering of the earlier tomato fruit clusters, we evaluated the effects of salt in fruit-set and fruit development. The total number of clusters per plant were quantified at flowering and ripening stages. In the first stage (between 8 to 9 wst), only plants treated with 160 mM NaCl showed a significant reduction (by 25%) in the number of clusters per plant relative to non-stressed plants (Fig. S2A). Likewise, in the case of clusters with ripe fruits at 14 wst, plants treated with 120 and 160 mM NaCl showed reductions of 27 and 50% compared to control treatment, respectively (Fig. S2B). No differences in the number of initial flowers from first and second truss between salt-treated and control plants were found, but fruit retention decreased by 48% in 160 mM NaCl treated-plants, measured as the number of fruits that did not abscise at 14 weeks of saline irrigation (Fig. S3).

**Figure 3:**
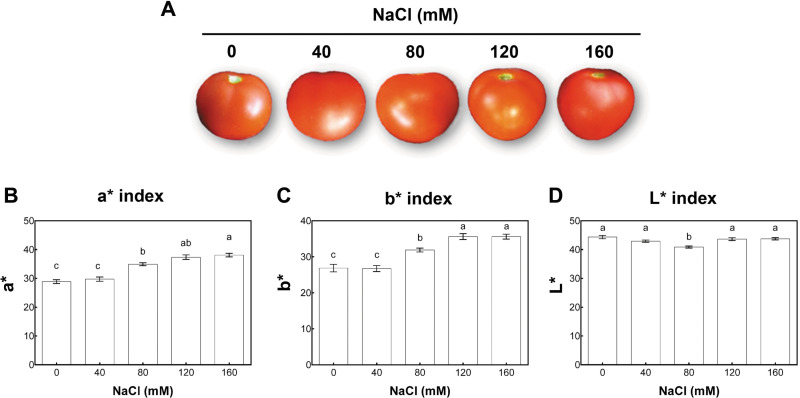
Colorimetric analysis of fruits from salt-stressed plants. (A) Visual analysis of tomato fruits obtained under the indicated NaCl irrigation regimes. (B, C, D) Colorimetric analysis of tomato fruits using the CIEL*a*b* system. Fruits were collected at 60 days post-anthesis (dpa) from plants exposed to 14 weeks of 0, 40, 80, 120, or 160 mM NaCl irrigation regimes. Data points represent mean ± SEM (three biological replicates). Different letters indicate statistically significant differences among treatments as determined by Tukey’s multiple comparison test (*P* ≤ 0.05). a*, a chromatic descriptor indicating red intensity; b*, a chromatic descriptor indicating yellow intensity; and L*, a chromatic component representing relative darkness or lightness.

According to the USDA color classification chart (USDA, 1976), fruit quality was analyzed at two developmental stages. These were defined in control conditions as matured-green (at 40 days post-anthesis (dpa) from plants with 11 wst) and red-ripe (at 60 dpa from plants with 14 wst). For each saline condition tested, matured-green and red-ripe developmental stages were defined at 40 and 60 dpa. We chose to use a dpa-based approach since a color-based strategy could be influenced by the salinity treatments. Since no visual differences based on the color classification chart were observed at 60 dpa between fruits from control or different salt-treatments, we evaluated the color of the fruit surface at the red-ripe developmental stage (60 dpa) using the CIEL*a*b* system based on the Opponent-Color Theory. In this procedure, the color is represented as three values or indexes: L* for the lightness from black (0) to white (100), a* from green (−) to red (+), and b* from blue (−) to yellow (+) (CIE, 2004). Regarding the a* index color parameter (that is indicative of red color intensity), tomato fruits from salt-stressed plants irrigated with 80, 120, and 160 mM NaCl presented higher values than the control plants. No significant differences compared to non-stressed plants were observed when a moderate salt concentration (40 mM NaCl) was used (Fig. 3). The salinity effect on b* index (indicative of yellow color intensity) was similar to the effect observed in a* values. On the contrary, the L* index was only significantly reduced in tomato fruits from plants under 80 mM NaCl treatment (Fig. 3). On the aggregate, these findings indicate that the salinity conditions (80 mM NaCl and over) increased red and yellow pigments in a concentration-dependent manner, suggesting an increase in the carotenoids level.

Compared with the non-treated control plants, fruit fresh weight (FW) significantly decreased (by around 50%) upon salinity treatments in plants irrigated with 120 and 160 mM NaCl. No significant changes in FW of tomato fruits from plants grown under moderate stress were observed (40 mM NaCl; Fig. S4A). Fruit caliber significantly decreased in all salt treatments (Fig. S4B). Contrary to the pattern of variation of FW and fruit diameter, total soluble solid content increased in plants under salt conditions. As shown in Fig. S4C, increases of around 28 and 47% were observed under 40 and 160 mM NaCl treatments, respectively.

### Salinity-induced differential expression of carotenoid-related genes and accumulation of metabolites in tomato fruits

**Figure 4:**
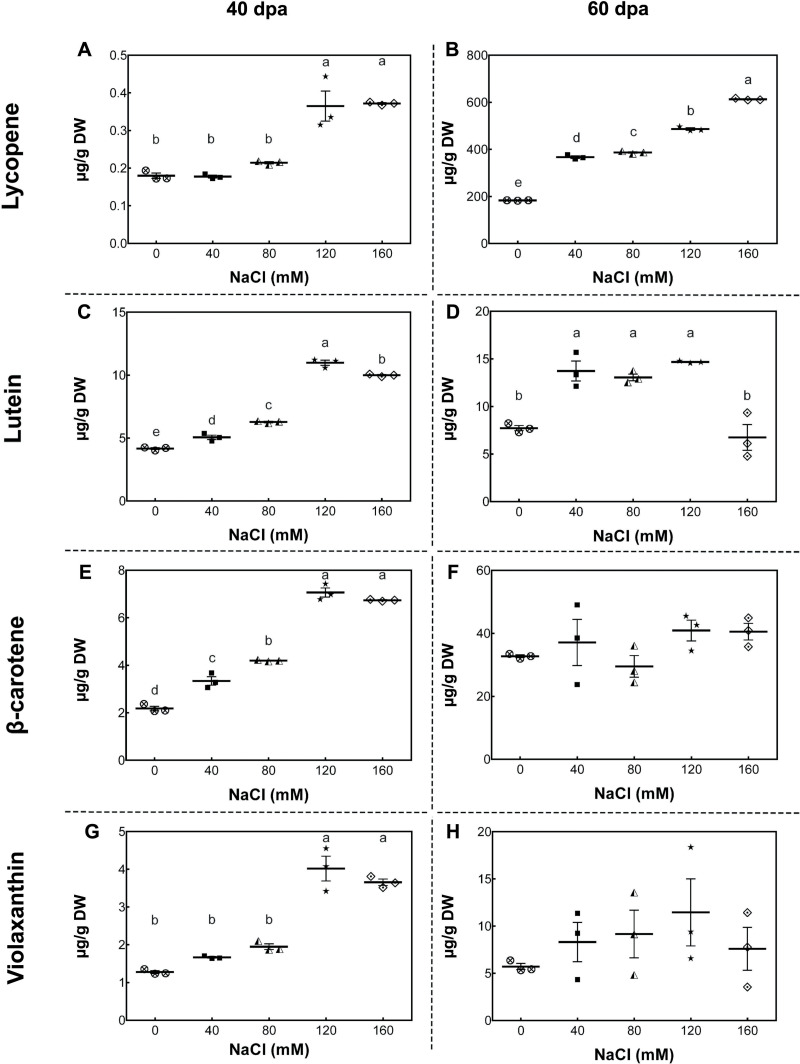
Carotenoid accumulation in tomato fruits from salt-stressed plants. (A, B) Lycopene; (C, D) Lutein; (E, F) β-carotene; (G, H) Violaxanthin. Fruit samples were collected at 40 and 60 days post-anthesis (dpa) from plants exposed for 11 and 14 weeks, respectively, employing the indicated NaCl irrigation regimes. Data points represent mean ± SEM (*n* = 3). Different letters indicate statistically significant differences among treatments, as determined by Tukey’s multiple comparison test (*P* ≤ 0.05).

To study the changes in carotenoid concentrations, we performed high-performance liquid chromatography (HPLC) analysis of fruits from plants exposed to different salinity treatments in the developmental stages described above. In mature-green fruits (40 dpa, 11 wst), lycopene content significantly increased (by more than 50%) in fruits from plants treated with 120 and 160 mM NaCl compared to fruits from control plants. In red-ripe fruits (60 dpa, 14 wst), a significant increase in lycopene levels was also observed, but from 40 mM NaCl upwards (Fig. 4). In the case of lutein at 40 dpa, all salt treatments resulted in a significant increase, but the greatest lutein concentration reached a peak at 120 mM NaCl. In the red-ripe stage (60 dpa, 14 wst), fruits from salt-treated plants also showed higher lutein concentration but not when 160 mM NaCl was used (Fig. 4). The β-carotene and violaxanthin contents showed significant differences between fruits from salt and control treatments only at 40 dpa (11 wst). In both cases, the higher carotenoid concentration was also reached in fruits from plants exposed to 120 and 160 mM NaCl (Fig. 4). This latter result shows a significant effect of the two most severe salt treatments in different carotenoid contents in tomato fruits. These findings are in agreement with the total carotenoid quantification (Fig. S5) since the highest levels were observed in fruits from plants treated with either 120 or 160 mM NaCl. Overall, increasing salinity concentration in our experimental conditions led to an increase in total carotenoid levels as well as the concentration of β-carotene and lycopene in mature-green (40 dpa) and red-ripe (60 dpa) tomatoes, respectively. Since this data was normalized to tomato dry weight (DW), our findings suggest that the increase in carotenoid levels might be caused by a plant physiological or stress response, rather than being the result of a decreased water uptake by fruits.

**Figure 5:**
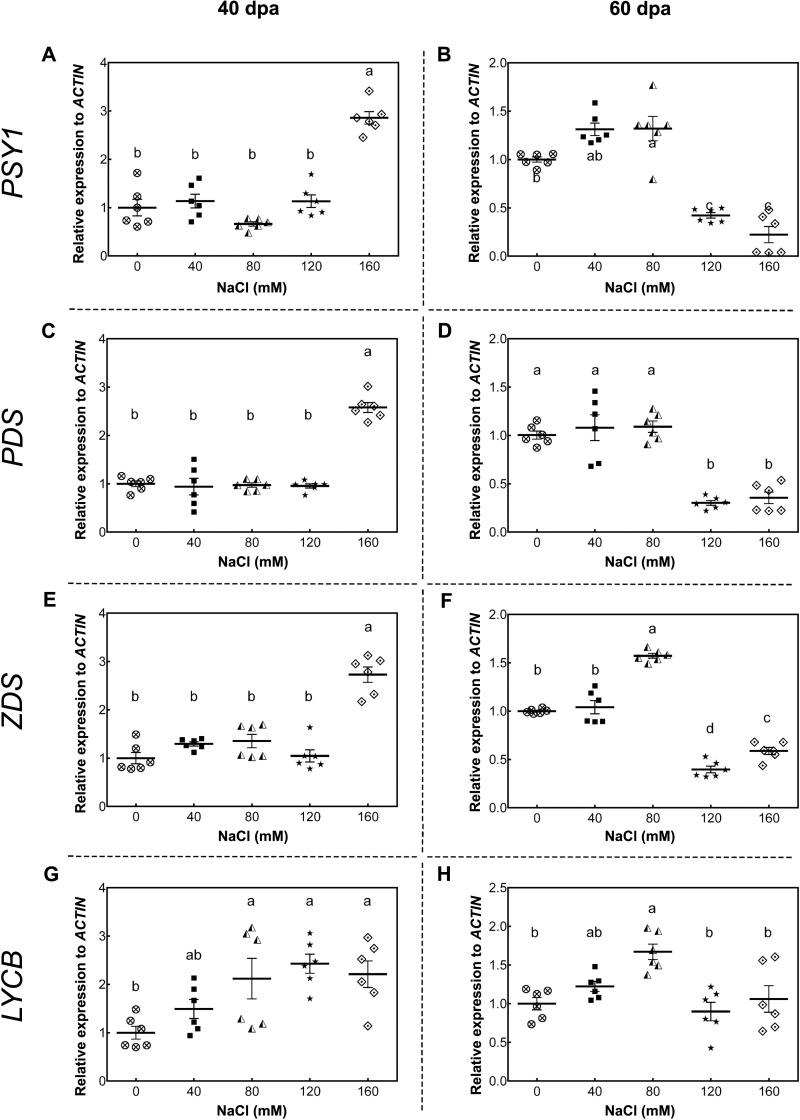
RT-qPCR analysis of key carotenoid biosynthesis pathway genes in response to salt stress. (A, B) *PSY1*; (C, D) *PDS*; (E, F) *ZDS*; (G, H) *LYCB*. Fruit samples were collected at 40 and 60 days post-anthesis (dpa) from plants exposed for 11 and 14 weeks, respectively, employing the indicated NaCl concentrations. Data points represent mean ± SEM (*n* = 6). Different letters indicate statistically significant differences among treatments, as determined by Tukey’s multiple comparison test (*P* ≤ 0.05).

As a starting point to understand this saline effect, we tested the gene expression of key carotenoid-pathway genes of tomato, such as phytoene synthase 1 (*PSY1*, *Solyc03g031860*), phytoene synthase 2 (*PSY2, Solyc02g081330*), phytoene desaturase (*PDS*, *Solyc03g123760*), *ζ*-carotene desaturase (*ZDS, Solyc01g097810*), lycopene β-cyclase (*LYCB, Solyc04g040190*), lycopene ε-cyclase (*LYCE*, *Solyc12g008980*) and carotenoid isomerase (*CRTISO*, *Solyc10g081650*), in order to determine whether changes in carotenoid content of tomato fruits upon salt stress was the result of a plant transcriptional response to high salt level exposure. In green fruits (40 dpa, 11 wst), *PSY1*, *PDS,* and *ZDS* showed a higher transcript accumulation in response to 160 mM NaCl, but no differences between control and lower salt-treated plants were observed (Fig. 5). Conversely, *PSY1*, *PDS,* and *ZDS* transcripts decreased in fruits from plants exposed to severe salt concentrations (120 and 160 mM) in red-ripe tomatoes (60 dpa, 14 wst). In the case of *LYCB*, its expression increased from 80 mM upwards compared to control treatment in green fruits (40 dpa, 11 wst), while in red-ripe fruits (60 dpa, 14 wst) an increased expression level was only observed in fruits from plants treated with 80 mM of salt (Fig. 5). Salinity treatment did not affect the gene expression of *LYCE, CRTISO,* and *PSY2* (Fig. S6).

Since all these genes are differentially expressed depending on the stage of fruit development, we analyzed the ripening-related ethylene genes 1-aminocyclopropane-1-carboxylate oxidase 1 (*ACO1*, *Solyc07g049530*), 1-aminocyclopropane-1-carboxylic acid synthase 2 (*ACS2*, *Solyc01g095080*), ethylene-inducible *CTR1*-like protein kinase (*CTR1*, *Solyc10g083610*), *CTR1*-like protein kinase 4 (*CTR4*, *Solyc10g085570*) and three ethylene receptors (*ETR3*, *Solyc09g075440*; *ETR4*, *Solyc06g053710*; *ETR6*, *Solyc09g089610*) in mature-green (40 dpa, 11 wst) and red-ripe (60 dpa, 14 wst) fruits from control and salt-stressed tomato plants. As observed in Fig. S7, no significant differences were determined in the gene expression of these ripening-related genes upon the salinity treatment. These results suggest that the salinity treatment has an influence on the carotenoid pathway in tomato fruit but no impact on the major ethylene-related ripening genes that modulate diverse maturation processes.

## Discussion

Salinity has been described as one of the most severe abiotic stress conditions affecting crop productivity in agricultural fields. This confines plant growth through different mechanisms, limiting the cropland available for cultivation (Orsini et al., 2010; Yao et al., 2011). Besides the salt-induced effect in vegetative growth, salinity also has a significant negative economic impact as it affects fruit development. Importantly, these effects on the stress response capacity of tomato are cultivar-dependent (Juan et al., 2005; Coesel et al., 2008), raising the need for further analyses to determine general responses of this model plant species to salt stress.

This work provides insights into the relationship between the response of vegetative organs and fruits of tomato plants to saline stress. Our results suggest a major relationship between the photosynthetic plant response, determined in vegetative tissue, and the plant fruit productivity and quality attributes—mainly carotenoids content—as a response to high-salt stress irrigation. In addition to the well-known negative effects on fruit yield (see below), this response also involves an impaired photosynthetic capacity with an increased content of beneficial carotenoids in fruits, which correlates with an upregulation of major genes of the carotenoid biosynthesis pathway.

One of the main plant features that can be affected by salinity is the photosynthetic capacity, which is in turn related to the decrease of plant growth and fruit yield (Munns, James & Läuchli, 2006; Chaves, Flexas & Pinheiro, 2009). In our study, we confirmed that the photosynthetic capacity of plants (Pn and gs) is negatively affected by salinity, showing an exposure time- and concentration-dependent impairment. A representative image of a plant in each salinity treatment at the end of the experiment is shown in Fig. S8. Moreover, as demonstrated here, the increase in NPQ indicates that even the lowest salinity levels tested here induced oxidative damage in the plant. Previous studies have described an up-regulation of NPQ in salt-sensitive cultivars exposed to moderate salinity (Zhang et al., 2012; El-Hendawy et al., 2017). In contrast, in the case of salt-tolerant cultivars, both ΦPSII and NPQ only started to change at the highest salinity condition (El-Hendawy et al., 2017). Consistent with this impaired photosynthetic capacity, the cv. Micro-Tom tomato plants used in this study showed chlorosis and a reduction in the shoot size (Fig. S8), as observed in previous studies carried out with commercial tomato genotypes, where salinity reduces biomass and shoot growth (Albacete et al., 2008; Dela Torre-González et al., 2017).

In the case of fruits, increasing salinity resulted in both negative and positive effects. For instance, a reduction of both the number of clusters per plant and the number of fruits per cluster was observed, with a detriment on tomato yield, as described in other studies (Adams & Ho, 1989; Adams, 1991; De Pascale et al., 2001). Nevertheless, we also observed an improvement in some quality traits. While salinity treatment lead to a reduction in fruit fresh weight and caliber, these fruits displayed a higher content of total soluble solids, which positively affected their organoleptic quality. These contradictory effects seem to depend on the cultivar and the ability of plants to tolerate salt stress (Juan et al., 2005; Coesel et al., 2008). Using high-salinity treatments, Yin et al. (2010) described that tomato Micro-Tom fruits obtained from plants under saline stress conditions showed a 1.6-fold increase in Brix° and a fresh weight reduction to 62% of the control plants. While these and our results can be interpreted as a consequence of a concentration-dependent response due to a reduction in the fruit’s volume as well as a lower water content, our results support an alternative explanation (see below). These authors proposed a salinity-induced stimulation of the accumulation of starch during early fruit development, enhancing soluble solids content in ripe fruits (Yin et al., 2010). Other independent studies have shown that higher dry matter, sugar, and organic acid concentrations can be observed upon increasing salinity treatments (Wu & Kubota, 2008; Van Meulebroek et al., 2016). Also, there may be a correlation of the aforementioned effects on tomato fruits, the decreased photosynthesis capacity, and a change in biomass partitioning between different sink tissues as a result of salt stress. Indeed, in tomato ’Moneymaker’ plants, the sink-related cell wall invertase enzyme, that regulates sucrose transport (Roitsch et al., 2003), has been previously reported as decreased in activity in leaves, together with an increased activity in roots (Albacete et al., 2008).

As reported herein, total carotenoids and lycopene content increased from moderate salt conditions. In the case of mature green tomato fruits, we also observed lutein, β-carotene, and violaxanthin as increased only under high salt-stress conditions. Initially, some studies attributed this improvement to a concentration effect as a result of decreased water uptake by fruits (Shi & Maguer, 2000; Dumas et al., 2003; Krauss et al., 2006). Nevertheless, our results and other studies strongly suggest that this higher carotenoid content might be activated/explained by a physiological effect response. As such, the increase in lycopene levels (2- to 3-fold change) was significantly higher than the increase in total soluble solid content (1.2- to 1.4-fold change). These results allowed us to discard, at least in part, the concentration effect. In agreement with our results, Borghesi et al. (2011) showed that lycopene and β-carotene concentration were enhanced by salinity in four different commercial tomato cultivars. De Pascale et al. (2001) and Wu & Kubota (2008) reported that moderate salinity (40–60 mM) had a positive effect on lycopene accumulation, while Massaretto et al. (2018) found that lycopene concentration increased under high salt stress (100 mM) in both a commercial cultivar and a tomato landrace. On the contrary, a negative or marginal effect on carotenoid accumulation was reported under salt stress (Dorais et al., 2000; Krauss et al., 2006; Van Meulebroek et al., 2012; Van Meulebroek et al., 2016; Ehret et al., 2013). Even when carotenoids content variation in response to salinity seem to correspond to a cultivar-dependent characteristic, it might be related to the ability of the plant to cope with stress and oxidative damage. Salinity causes damage to the photosynthetic apparatus, which might prevent proper energy dissipation (Demmig-Adams & Adams, 1992), and consequently, reactive oxygen species (ROS) synthesized as a stress by-product. Hence, plants must counteract the damages through the synthesis of antioxidant molecules, such as carotenoids, as a plant non-enzymatic mechanism for the detoxification of ROS (Havaux & Niyogi, 1999; Abogadallah, 2010).

Besides the phenotypic and physiological responses to salt stress, we also studied the expression of some key carotenoid-pathway genes in fruits. To our knowledge, only a few studies in response to salt stress include a gene expression analysis. Moreover, the relationship between gene expression regulation and the accumulation of carotenoids in response to salinity had not been described (Ehret et al., 2013). As reported herein, the accumulation of carotenoids is not exclusive to mature fruits. In non-ripe fruits, we detected increased *PSY1*, *PDS,* and *ZDS* transcript levels when employing the highest saline treatment, while lycopene, whose synthesis occurs after the activity of the enzymes encoded by these genes, increased from 120 mM NaCl onwards. On the other hand, ripe fruits showed increased transcript levels at lower salt concentrations, but higher lycopene content in all employed saline conditions, differentially increasing as salinity level rises. The differential transcripts levels might be related to a modified stress response of fruits depending on its developmental stage, although we did not observe a direct correlation between changes in gene expression and concentration of metabolites. Rienth et al. (2014) described that anthocyanin-relative transcript levels are differentially affected by heat stress depending on daytime treatment and berry developmental stages. Variations in the carotenoid contents in response to salinity might not be only due to changes in expression of the carotenoid biosynthetic pathway genes but also to post-transcriptional regulation and/or modification in these metabolite storage capacities in the plant, as observed in response to light (reviewed by Llorente et al., 2017). Recently, several post-transcriptional and post-translational regulatory mechanisms had been described in carotenoid pathways, such as protein modifications, protein and metabolite storage/degradation, and feedback regulation by end products. Arabidopsis PSY enzyme was shown to be activated by Orange (Or) protein (Zhou et al., 2015) and feedback-regulated by 5′-UTR translational elements in PSY mRNA (Álvarez et al., 2016). In addition, PSY and PDS enzymes are regulated at post-transcriptional level by the redox state of electron acceptors in membrane chromoplasts of *Narcissus pseudonarcissus* (Welsch et al., 2000). Another example is a feedback-regulation by signal molecules derivate of apocarotenoid. Carotenoids are degraded by carotenoid cleavage dioxygenases (CCDs) and 9-cis-epoxycarotenoid dioxygenases (NCEDs) and produce apocarotenoids. Avendaño Vázquez et al. (2014) identified an apocarotenoid-derived signal in Arabidopsis leaf that regulates *PSY* gene and other chloroplast- and nuclear-encoded genes.

## Conclusions

Salinity impacts both vegetative and reproductive growth in tomato plants, hampering photosynthetic capacity, increasing induction of key carotenoid-related genes and carotenoid levels, thus suggesting a key relationship between plant photosynthetic response and yield.

##  Supplemental Information

10.7717/peerj.9742/supp-1 Figure S1 Maximum quantum yield of PSII (Fv/Fm) in salt-stressed plants Samples were collected at (A) 2 and (B) 8 weeks of 0, 40, 80, 120, or 160 mM NaCl irrigation. Data points represent mean ± SEM of five and between three and five biological replicates for 2 and 8 weeks of salinity treatment (wst), respectively. Different letters indicate statistically significant differences among treatments, as determined by Tukey’s multiple comparison test (*P* ≤ 0.05)

10.7717/peerj.9742/supp-2 Figure S2 Analysis of tomato clusters in salt-stressed plants Plants were evaluated at 9 (A) and 14 (B) weeks, employing the indicated NaCl concentrations. Data points represent mean ± SEM (*n* = 3). Different letters indicate statistically significant differences among treatments as determined by Tukey’s multiple comparison test (*P* ≤ 0.05).

10.7717/peerj.9742/supp-3 Figure S3 Influence of salinity in tomato fruit production (A) The number of initial flowers and (B) the number of remaining fruits in plants exposed for 14 weeks employing the indicated NaCl concentrations. Data points represent mean ± SEM (*n* = 3). Different letters indicate statistically significant differences among treatments, as determined by Tukey’s multiple comparison test (*P* ≤ 0.05).

10.7717/peerj.9742/supp-4 Figure S4 Tomato fruit quality parameters in salt-stressed plants (A) Fruit fresh weight; (B) Fruit caliber; (C) Soluble solids. Fruit samples were collected at 40 and 60 days post-anthesis (dpa) from plants exposed to salinity treatment for 11 and 14 weeks, respectively, employing the indicated NaCl concentrations. Data points represent mean ± SEM (*n* = 3). Different letters indicate statistically significant differences among treatments as determined by Tukey’s multiple comparison test (*P* ≤ 0.05).

10.7717/peerj.9742/supp-5 Figure S5 Total carotenoid content of tomato fruits from salt-stressed plants Fruit samples were collected at 40 (A) and 60 (B) days post-anthesis (dpa) from plants exposed for 11 and 14 weeks, respectively, employing the indicates NaCl concentrations. Data points represent mean ± SEM (*n* = 3). Different letters indicate statistically significant differences among treatments as determined by Tukey’s multiple comparison test (*P* ≤ 0.05). DW, dry weight.

10.7717/peerj.9742/supp-6 Figure S6 RT-qPCR analysis of carotenoid biosynthesis pathway genes in response to salt stress (A, B) *LYCE*; (C, D) *CRTISO*; (E, F) *PSY2*. Fruit samples were collected at 40 (A, C, E) and 60 (B, D, F) days post-anthesis (dpa) from plants exposed for 11 and 14 weeks, respectively, employing the indicates NaCl concentrations. Data points represent mean ± SEM (n=6). Different letters indicate statistically significant differences among treatments, as determined by Tukey’s multiple comparison test (*P* ≤ 0.05).

10.7717/peerj.9742/supp-7 Figure S7 RT-qPCR analysis of ripening-related ethylene genes in response to salt stress

10.7717/peerj.9742/supp-8 Figure S8 Representative plants in salinity treatments A representative plant at 14 weeks after salinity treatments. The concentration of NaCl is indicated in each pot. The white line represent 10 cm.

10.7717/peerj.9742/supp-9 Table S1 Primer sequences used in RT-qPCR analysis

10.7717/peerj.9742/supp-10 Data S1 Raw data for all figures and supplemental figures

## Additional Information and Declarations

### Data Availability

The following information was supplied regarding data availability: The raw data is available in the Supplemental File.
