# Supplementary figures and images for: Salinity impairs photosynthetic capacity and enhances carotenoid-related gene expression and biosynthesis in tomato (Solanum lycopersicum L. cv. Micro-Tom)
Source: PeerJ. 2020 Sep 17;8:e9742. doi: 10.7717/peerj.9742 (PMC7502237; doi:10.7717/peerj.9742)

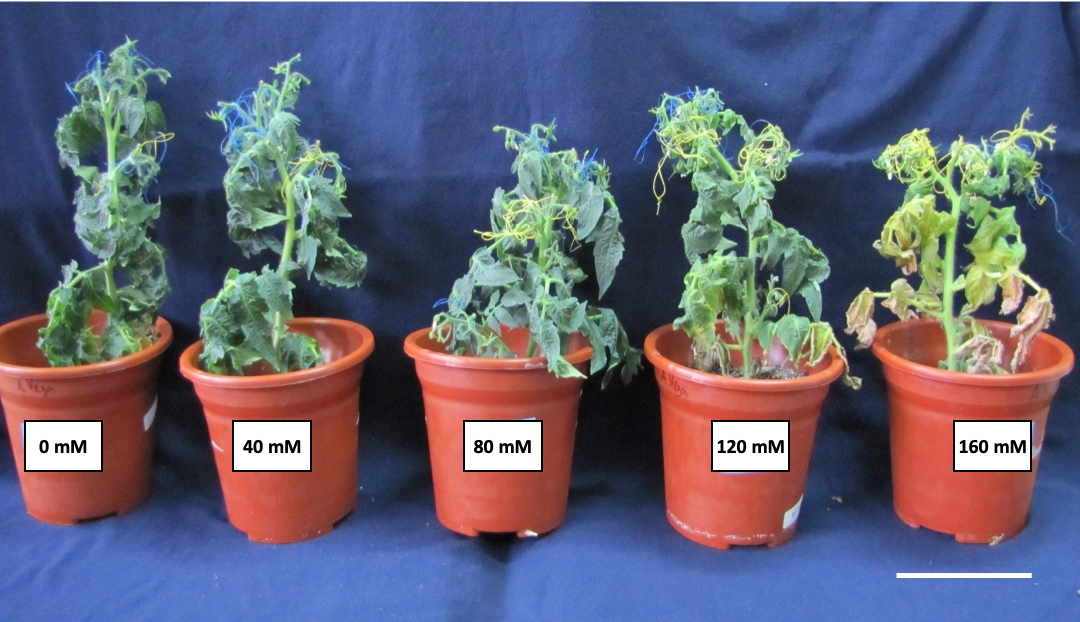

Supplement: Figure S8 — A representative plant at 14 weeks after salinity treatments. The concentration of NaCl is indicated in each pot. The white line represent 10 cm. [file peerj-08-9742-s008.png]
