# Supplementary material for: Salinity impairs photosynthetic capacity and enhances carotenoid-related gene expression and biosynthesis in tomato (Solanum lycopersicum L. cv. Micro-Tom)
Source: PeerJ. 2020 Sep 17;8:e9742. doi: 10.7717/peerj.9742 (PMC7502237; doi:10.7717/peerj.9742)
Supplement: Table S1 [file peerj-08-9742-s009.docx]

| **Gene** | **ID** | **Primer sequence (5’ 🡪 3’)** | |
| --- | --- | --- | --- |
| ***ACO1*** | *Solyc07g049530* | Forward | ATGCGCCACTCTATTGTGGT |
|  |  | Reverse | TCCCGTCTGTTTGCGCAATTAC |
| ***ACS2*** | *Solyc01g095080* | Forward | TCTCGCCTGGATCTTCGTTTGA |
|  |  | Reverse | CAACACCTACGAACCTCCGAAT |
| ***CRTISO*** | *Solyc10g081650* | Forward | CGAGATCGCCAAATCCTTAGCA |
|  |  | Reverse | AAGCTTCACTCCCACAGCTTT |
| ***CTR1*** | *Solyc10g083610* | Forward | CAGCTGTCGGCTTTAACAGGAA |
|  |  | Reverse | TCATTAGCCCAGCAAGCCTCAA |
| ***CTR4*** | *Solyc10g085570* | Forward | TGGAATGGCTCTGATGTTGCTG |
|  |  | Reverse | TGGATGTCGCAACCGCTTCATA |
| ***LYCb*** | *Solyc04g040190* | Forward | TCAGAGAGTCGTTGGAATCGGT |
|  |  | Reverse | AACAGGAGCCGCAGCTAGT |
| ***LYCe*** | *Solyc12g008980* | Forward | TGGTCTTACATACCGGTTGGTG |
|  |  | Reverse | TGTGGCTGGATGAACCATGCTA |
| ***ETR3*** | *Solyc09g075440* | Forward | GGCTTGCCATTTGCAGACGATT |
|  |  | Reverse | ACAAACGTGACAGTGGTTCCCT |
| ***ETR4*** | *Solyc06g053710* | Forward | GCTGGTTCAGTTGATGCAAGGA |
|  |  | Reverse | AATTGATGGCCGCAGTTGAAGC |
| ***ETR6*** | *Solyc09g089610* | Forward | GACAGACCTGGCCTTTGATCAT |
|  |  | Reverse | GGCGAGTCCTGATAAGAGCAAT |
| ***PDS*** | *Solyc03g123760* | Forward | CTTCAATGGAAGGCGCTGTCT |
|  |  | Reverse | ACGCTTGCTTCCGACAACTTCT |
| ***PSY1*** | *Solyc03g031860* | Forward | ATTTGCTGGAAGGGTGACCGAT |
|  |  | Reverse | GCTCAATTCTGTCACGCCTTTC |
| ***PSY2*** | *Solyc02g081330* | Forward | GGACGAGATTGAAGCAAACGAC |
|  |  | Reverse | TGCATAAGCAATGGGCAACGTC |
| ***ZDS*** | *Solyc01g097810.3* | Forward | TTGGAGCGTTCGAGGCAATTGA |
|  |  | Reverse | GCCAATGCAAGATCTGCAAAGC |
| ***ACTIN*** | *Solyc04g011500.3* | Forward | GAAATAGCATAAGATGGCAGACG |
|  |  | Reverse | ATACCCACCATCACACCAGTAT |
